# Powder Metallurgy Processing of a W_x_TaTiVCr High-Entropy Alloy and Its Derivative Alloys for Fusion Material Applications

**DOI:** 10.1038/s41598-017-02168-3

**Published:** 2017-05-16

**Authors:** Owais Ahmed Waseem, Ho Jin Ryu

**Affiliations:** Department of Nuclear and Quantum Engineering, Korea Advanced Institute of Science and Technology, 291 Daehakro, Yuseong-gu, Daejeon 34141 Republic of Korea

## Abstract

The W_x_TaTiVCr high-entropy alloy with 32at.% of tungsten (W) and its derivative alloys with 42 to 90at.% of W with *in-situ* TiC were prepared via the mixing of elemental W, Ta, Ti, V and Cr powders followed by spark plasma sintering for the development of reduced-activation alloys for fusion plasma-facing materials. Characterization of the sintered samples revealed a BCC lattice and a multi-phase structure. The selected-area diffraction patterns confirmed the formation of TiC in the high-entropy alloy and its derivative alloys. It revealed the development of C15 (cubic) Laves phases as well in alloys with 71 to 90at.% W. A mechanical examination of the samples revealed a more than twofold improvement in the hardness and strength due to solid-solution strengthening and dispersion strengthening. This study explored the potential of powder metallurgy processing for the fabrication of a high-entropy alloy and other derived compositions with enhanced hardness and strength.

## Introduction

Nuclear fusion-based power reactors require advancements with regard to high-temperature and plasma-facing materials^1^ in order to prevent degradation due to the exposure to high-temperature and high-energy neutron flux^2^ produced during deuterium-tritium fusion reactions^3^. The severity of the service environment is expected to increase in upcoming fusion reactors with increased power ratings and those under transient scenarios^4^. For instance, the heat load on the divertor, a key component of the tokamak design^3, 5^, is expected to increase to 10 MW/m^2^ (steady state) and 20 MW/m^2^ (transient for 10 seconds)^4^. The inner and outer vertical targets of the divertor, where the kinetic energy of plasma particles is converted into heat^6^, must withstand loads up to ~7–40 MJ/m^2^ and ~4–25 MJ/m^2^, respectively, under plasma disruption^4, 7^. Therefore, unprecedented thermal, mechanical and physical characteristics are required for fusion reactor materials^8^.

The requirement of innovative plasma-facing and high-temperature materials^9^ has turned researchers’ attention towards refractory metals due to their high-temperature properties, such as a high melting temperature, good strength, and good creep resistance^10^. Intensive research has explored the properties and performance of tungsten in plasma-facing applications^2, 11, 12^. A unique combination of various physical, chemical, mechanical and irradiation characteristics, including a high melting temperature, good thermal conductivity, a proper sputtering threshold^13, 14^, good creep strength, a low level of tritium retention, and neutron activation and erosion^15–28^ have made W a potential material^29^ for plasma-facing components of fusion reactors^2, 30–32^.

Although the potential of W for use as a plasma-facing material is accepted, its commercial roles remain restricted due to its high ductile-to-brittle transition temperature (DBTT), radiation-induced embrittlement, its low yield strength and the fact that it oxidizes readily at elevated temperatures into WO_3_, which is volatile above ~1000 °C^17, 21, 21–24, 33–39^. Mitigation of these deficiencies is essential^15, 40^. Therefore, extensive research is being done to explore new W alloys with suitable properties^8, 41–46^. For example, several W-based binary alloys, including W-Re^42, 47–49^, W-Ta^50, 51^, W-V^44, 52^, W-Ti^53–55^, W-Mo^56, 57^ and W-Cr^55, 58, 59^ have been studied. Significantly improved engineering characteristics of these alloys, as compared to those of pure tungsten, have also been explored^44, 47, 48, 50, 53, 54, 56, 58^,. However, detailed analyses of conventional binary alloys revealed several constraints as well. For example, some W-based binary alloys showed brittle behavior^9, 37^, and certain alloys, such as W-Re^48, 52^ and W-Os^60^, underwent irradiation-induced embrittlement. The presence of metastable phases has also been found to deteriorate the mechanical properties of W-Ti and W-V (to a lesser extent than that found in W-Ti) binary alloys^52^. The difficulty associated with obtaining a perfect solid solution in W-V^61^, the grain growth^56^ and the reduced elongation (or ductility)^62^ associated with W-Mo alloys also hinder the utilization of W-based binary alloys in harsh service environments. The drawbacks of W alloys play a role in diverting researchers’ attention towards high-entropy alloys (HEAs) for the future development of W-rich materials for plasma-facing applications.

Contrary to conventional alloys, which consist of alloying element(s) added to one or two principal elements, HEAs contain five to thirteen equimolar or near-equimolar principal elements between 5 to 35 at.%; hence, the term “multi-principal component alloy” (MCA) is sometimes used^63–77^. Single-phase HEA is expected to form frequently due to its high mixing entropy^78–81^, but the microstructure of some HEAs typically shows two or more phases. Moreover, in contrast to the intermetallic structure commonly found in conventional alloys, HEAs exhibit simple BCC, FCC, HCP or a mixture of these crystal structures due to their good mutual solubility^77, 82–88^, such as WNbMoTa and WNbMoTaV^89, 90^, which show a single-phase BCC crystal structure. The purpose of incorporating multiple principal elements is to produce a high-entropy solid solution with improved engineering characteristics^79, 91^.

The excellent properties of HEAs, including their enhanced mechanical strength even at high temperatures along with improved wear and oxidation resistance, fatigue and high-temperature fracture resistance, good thermal stability and toughness^70–73, 75, 77, 80, 85, 87, 92–97^ reflect their potential for use in aerospace, nuclear^75, 82, 98^ and other industrial applications^84, 99^. Research for a further improvement of the behavior of HEAs via a thermomechanical treatment or by precipitation hardening and/or grain refinement is also in progress^69, 79^.

Al, Cr, Fe, Co, Ni, Cu. Ti, V and Mn have long been commonly used in HEA systems^10, 68, 69, 85, 88, 96–98, 100–103^. However, the use of refractory elements (W, Ti, Ta, Cr, V, Hf, Zr, Nb and Mo)^87^ to produce HEAs for high-temperature applications has increased, and superior mechanical properties such as yield strengths of 405 MPa and 477 MPa correspondingly at 1600 °C for WTaNbMo and WTaNbMoV, the 40% room-temperature compression strain of TaNbHfZrTi, and the 1.3 GPa compressive strength of CrNbTiZr and CrNbTiVZr^87, 104^ have been reported. Other research has investigated NbTiVTaAl_x_, CrNbTiVZr, NbCrMoTiAl_0.5_, NbCrMoVAl_0.5_, NbCrMoTiVAl_0.5_, NbCrMoTiVAl0.5Si_0.3_ and AlMo_0.5_NbTa_0.5_TiZr^80, 86^.

The research presented in this paper is novel in many respects, as a pioneering HEA is developed as per the compositional criteria which are adopted for developing reduced activation ferritic martensitic steels^105, 106^ (which can not only be used in fission applications (like other irradiation resistant HEA) but also in fusion plasma facing applications (explained in Supplementary Section S1)) is developed along with other W-rich alloys derived from HEA compositions by increasing the W content. In order to develop HEA for low neutron activation properties, tungsten (W) and tantalum (Ta) were chosen from the five most commonly used refractory metals (tungsten, tantalum, niobium, molybdenum and rhenium) owing to their adequate mechanical properties and resistance to irradiation-induced embrittlement and swelling^107^. Mo and Nb were restricted given their nuclear activation properties^107^. Re was also avoided in order to prevent the formation of irradiation-induced embrittling precipitates^48, 52^. Titanium (Ti) and vanadium (V), representing a broader definition of refractory metals, were also selected. Ti plays a significant role in improving the sintered density^44, 52–54^ through rapid and significant interdiffusion, mass transport through the interfaces, and rearrangements of particles^54, 108, 109^, whereas V aids in improving the strength and hardness of refractory HEAs^90^. Another refractory metal, chromium (Cr), was also chosen for use considering its ability to impart passivation^110^. The low neutron activation properties of W^111^, Ta^112^, Ti^113^, V^114^ and Cr^113^ also favor their selection for the development of materials for potential fusion plasma facing applications. The synthesis technique was selected considering its influence on the properties of the engineering materials^91^; e.g., alloys undergo segregation when prepared by melting^9^, and embrittlement and porosity are attributes of conventional sintering^115^, whereas the low thickness of the final product is a limit of the physical vapor deposition (PVD) approach^32^. The utilization of mechanical alloying (MA) to develop a HEA with enhanced properties has also been reported^32, 39, 42, 84, 91, 99, 100, 116–119^. However, no research has been reported thus far regarding the enhancement of the hardness and strength by the elemental powder mixing of refractory elements, which is a simpler, economical and less time-consuming technique than high-energy milling. Powder milling not only increases contamination due to wear of milling media^32, 42^ but it makes powders susceptible to oxidation as well^120^. This paper presents the potential of elemental powder mixing followed by spark plasma sintering (SPS) given the ability of this process to consolidate the powder into a fine-grained material with a controlled structure and high density while saving time due to the short soaking period and high heating and cooling rates^8, 17, 18, 119, 121, 122^, for the fabrication of a HEA and its derivative alloys for high-temperature and plasma-facing applications.

## Experimental

In this study, 99.9% pure elemental powders of W (1.21 µm from Daegu Tek. Co.), V (<75 µm from Kojundo Korea), Cr (63 µm from Kojundo Korea), Ta (<45 µm from Seo Gwang Metal) and Ti (45 µm from Kojundo Korea) were used. The powders were mixed to develop the mixture of alloys with 30 to 90 at.% W and equal amounts of the remaining elements, i.e., W_x_TaTiVCr, where x is the atomic fraction of W (0.3 to 0.9). The detailed nominal compositions are given in Table 1. Shaker mixing in a Turbula shaker-mixer was carried out in plastic vials at a speed of 30 rpm for up to three hours with a 1:1 ball-to-powder ratio. In order to fabricate pellet-shaped bulk samples, the powder mixtures were fed into graphite molds, and the mold surfaces were coated with boron nitride to avoid a severe interfacial reaction of the samples with the graphite. The molds were then covered with a 6–10-mm-thick carbon felt blanket to prevent heat loss^123^. Consolidation of the powder mixture was carried out by spark plasma sintering (SPS, Dr. Sinter SPS-515S, Japan) at 1600 °C and under a vacuum (10^−3^ torr) with an axial pressure of 50 MPa for ten minutes. The heating rate was kept higher, in this case at 100 °C/min. The optimization of experimental conditions in order to achieve improved diffusion and enhanced sintering has been described in Supplementary Information (Section S2).

**Table 1:** Nominal and real composition of W_x_TaTiVCr samples (in at.%).

	Powder Mixtures (_p_)		Sintered Samples (_s_)	
	Name (*xW_p_)	Nominal Samples	Name (*xW_s_)	Real Samples
HEA	30 W_p_	W_0.3_(TaTiCrV)_0.7_	32 W_s_	W_0.32_Ta_0.18_Ti_0.18_V_0.20_Cr_0.19_
HEA derivatives	40 W_p_	W_0.4_(TaTiCrV)_0.6_	42 W_s_	W_0.42_Ta_0.15_Ti_0.14_V_0.14_Cr_0_._14_
	50 W_p_	W_0.5_(TaTiCrV)_0.5_	56 W_s_	W_0.56_Ta_0.15_Ti_0.09_V_0.11_Cr_0.09_
	60 W_p_	W_0.6_(TaTiCrV)_0.4_	63 W_s_	W_0.63_Ta_0.09_Ti_0.09_V_0.09_Cr_0.09_
	70W_p_	W_0.7_(TaTiCrV)_0.3_	71 W_s_	W_0.71_Ta_0.04_Ti_0.07_V_0.07_Cr_0.07_
	80 W_p_	W_0.8_(TaTiCrV)_0.2_	77 W_s_	W_0.77_Ta_0.05_Ti_0.07_V_0.05_Cr_0.06_
	90 W_p_	W_0.9_(TaTiCrV)_0.1_	90 W_s_	W_0.90_Ta_0.03_Ti_0.02_V_0.03_Cr_0.02_

The actual composition of the samples was determined by inductive coupled plasma optical emission spectrometry (ICP-OES, iCAP 6300 Duo, Thermo Scientific Co., UK) and by a gas fusion analysis (ONH-2000 & CS-2000, ELTRA, Germany). The density of the sintered samples was determined by the Archimedes’ method. The effects of varying the composition on the crystallographic and microscopic features were examined by subjecting the samples to x-ray diffraction (XRD, D/MAX-2500, RIGAKU, USA), scanning electron microscopy - energy dispersive spectroscopy (SEM-EDS, FEI Magellan 400, USA) and transmission electron microscopy (JEM2100F, JEOL Ltd., Japan). The compositions of several phases observed via SEM were determined through an electron probe microanalysis (EPMA 1610, AE11, Shimadzu, Japan).

The pellet-shaped samples were cut from the center into two halves to expose the cross-section, on which a micro-Vickers hardness (402MVD, Wolpert Wilson Instruments, Germany) test with a 1 kg load and a 10-second dwell time was carried out to assess the mechanical behavior of the materials. Cylindrical samples 6 mm high produced by wire cutting of the sintered pellets, with diameters of 4 mm, were subjected to a room-temperature compression test using an Instron 5982 machine with a strain rate of 10^−3^/s.

## Results and Discussion

The W_0.3_(TaTiCrV)_0.7_ sample showed improved relative density and microstructural homogeneity with the increasing sintering temperature from 1300 °C to 1600 °C. Sintering at 1600 °C produced a fully dense sample with good homogeneity, as shown in Fig. S2. Hence, 1600 °C was selected for sintering all W_x_TaTiVCr samples.

In order to determine the actual composition (after sintering) of the samples, inductively coupled plasma optical emission spectrometry (ICP-OES) was carried out. The results of a chemical analysis of the sintered samples are shown adjacent to the nominal compositions of the powder mixtures in Table 1.

The chemical analysis of the HEA (having near equiatomic composition) and the HEA derivatives (the W-based alloys derived from a near-equiatomic composition via gradually increasing the W content) revealed changes in the composition after sintering. Slight compositional inhomogeneity, which cannot be ruled out in a simply mixed powder, formed a number of highly localized binary and/or ternary compositions with a melting point in the sintering temperature range. The nominal compositions underwent variation due to a loss of the constituent elements by localized melting and partial leakage of the melt from the mold during sintering. Certain alloys based on Ti-Cr or Ti-Cr-V which melt at ~1400–1600 °C (depending on the ratio of the constituents)^124^ account for the change of the chemical composition of the sintered samples due to the fractional leakage of the melt during the sintering step. The 30 W_p_, 40 W_p_, 50 W_p_, 60 W_p_, 70 W_p_, 80 W_p_, and 90 W_p_ samples reached corresponding levels of 32 W_s_, 42 W_s_, 56 W_s_, 63 W_s_, 71 W_s_, 77 W_s_ and 90 W_s_ (Table 1). This nomenclature will be used in the following text.

Spark plasma sintering in graphite molds induces carbon in sintered samples^125–132^. Through a gas fusion analysis, carbon levels of 0.37 at.%, 0.53 at.%, 0.87 at.%, 0.20 at.%, 0.13 at.%, 0.11 at.%, and 0.2 at.% were determined in the 32 W_s_, 42 W_s_, 56 W_s_, 63 W_s_, 71 W_s_, 77 W_s_ and 90 W_s_ samples, respectively.

The calculated density of xW_s_ was estimated using the volume-averaged density of the constituent alloying elements, as determined by a chemical analysis. Along with some experimental fluctuations, an increasing trend in the relative density with a decrease in the W content was observed. Figure S3 (Supplementary Information) presents the measured density levels of the 32 W_s_, 42 W_s_, 56 W_s_, 63 W_s_, 71 W_s_, 77 W_s_, and 90 W_s_ samples.

The XRD patterns of the powder mixtures of xW_p_ and the sintered samples of xW_s_ are shown in Figure S4 (a) and (b), respectively. In Figure S4(a), the diffraction peaks of all constituents present in the alloy powder are clearly visible.

The higher configuration entropy of xW_s_, the valence electron concentration (VEC) of xW_s_ < 6.87^91^, and the negligible mixing enthalpy led to the development of a solid solution^70^ after sintering at 1600 °C and produced a high-entropy alloy with the body-centered cubic (BCC) crystal structure. The diffraction peaks of the samples sintered at 40°, 58° and 73° from the (110), (200), and (211) planes, respectively, confirm the presence of a BCC crystal structure, as observed previously in sintered refractory alloys^53^ and HEAs^103, 133^ due to the high degree of mutual solubility of their constituents^134, 135^. The presence of intermetallics in HEAs is well known^89, 93^; however, the low volume fraction of intermetallics prevents XRD patterns from giving any indication of their presence. Therefore, a TEM analysis (as presented in the following sections) is necessary to confirm the presence/absence of intermetallics in xW_s_. The comparison of the XRD patterns of the alloy samples in Figure S4(b) with the diffraction peaks of pure W sample reveals slight broadening of peaks due to microstresses^136^ arising from the variations in the composition^102^ (i.e., the decreasing W-content and increasing content of Ta, Ti, V and Cr in the xW_s_ samples).

**Figure 1:**
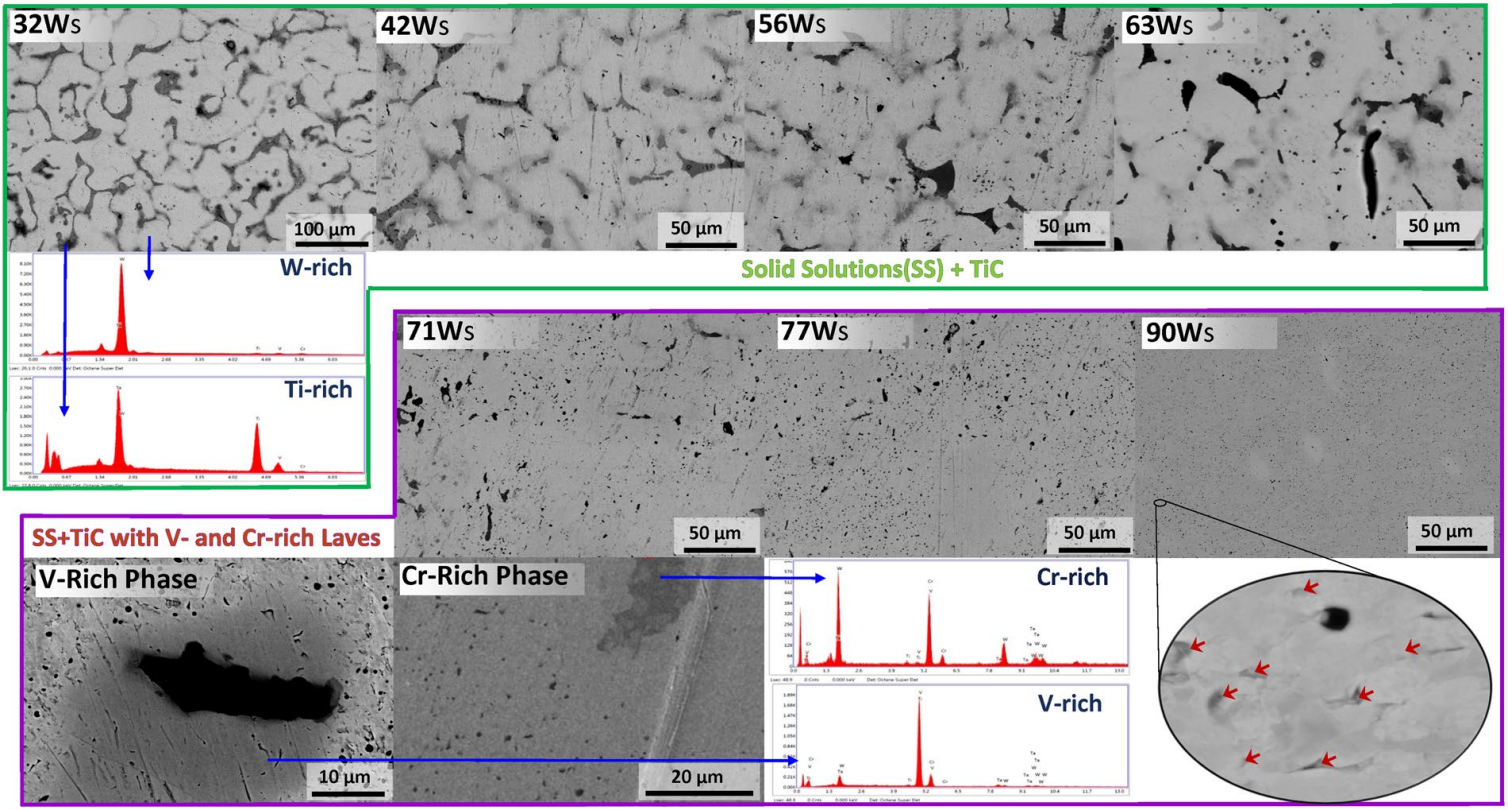
SEM micrographs of the xW_s_ samples sintered at 1600 °C.

The microstructures of the as-polished xW_s_ samples (HEA (x = 32) and its derivatives (x = 42 to 90)), captured with backscattered electrons, as shown in Fig. 1, exhibit randomly distributed phases with black, dark gray and light gray regions in a bright single phase HEA matrix. The chemical nature, analyzed via point energy-dispersive spectroscopy (EDS), revealed the enrichment of Ti and W in the dark and bright regions of the microstructures, respectively. No indication of a Ti-rich phase, which indicates a hexagonal-close-packed (HCP) crystal structure in a W-Ti alloy, was observed in the XRD patterns^53^.

**Figure 2:**
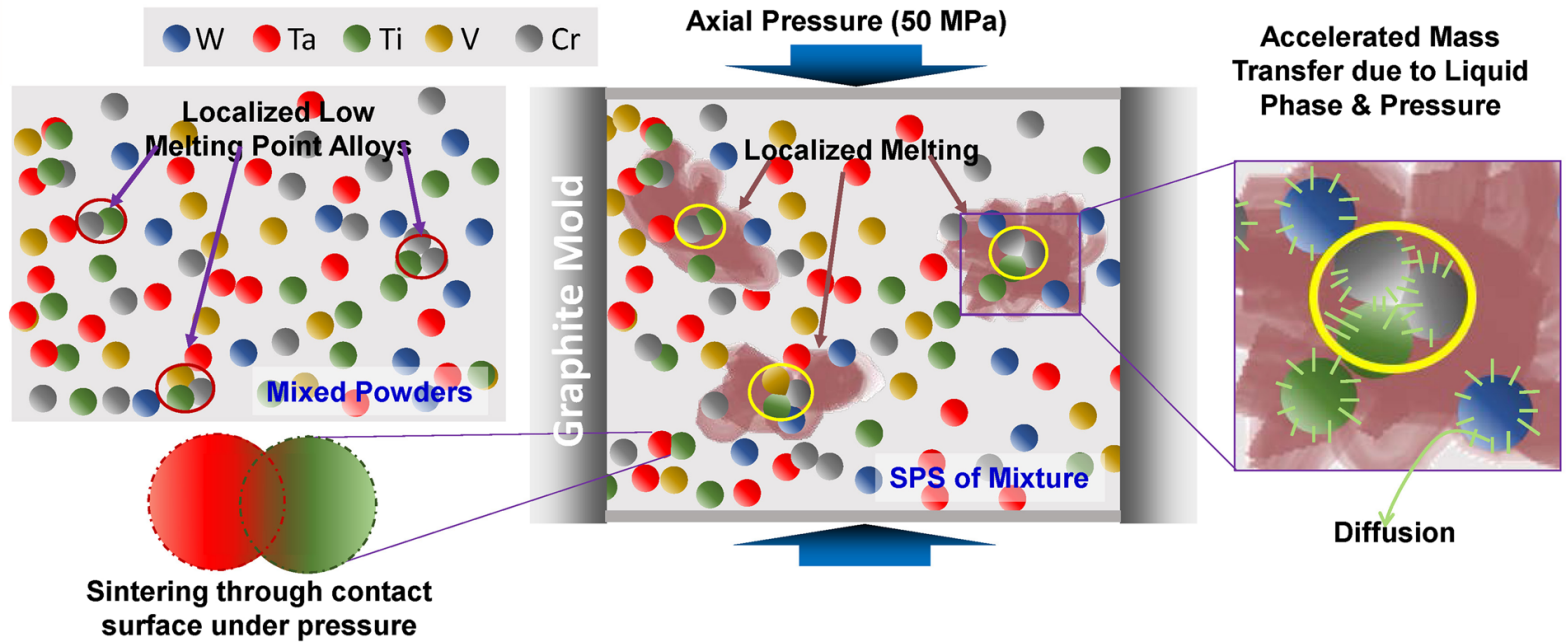
Illustration of sintering mechanism of W_x_TaTiVCr alloy system.

The SEM images of the 32 W_s_, 42 W_s_, 56 W_s_ and 63 W_s_ samples revealed large grains as compared to those which contain 71 to 90 at.% of W. The large grains stem from the liquid-phase assisted sintering due to the melting of the locally formed Ti-Cr- or Ti-Cr-V-based alloys having melting points of ~1400–1600 °C (depending upon the ratio of the constituents)^124^, which may also be present in simply mixed elemental powders at some highly localized points. The molten phase, rich in Ti, was found to accumulate along the grain boundaries in the microstructures of xW_s_ containing 32 to 63%W. The mechanism of sintering has been illustrated in Fig. 2 and described in Supplementary Section S3.

In addition, 70at.% of W in a nominal composition of W_x_TaTiCrV has been found to be a critical amount for suppressing liquid-phase-assisted sintering, as the 71 W_s_, 77 W_s_ and 90 W_s_ samples underwent solid-state dominant sintering with no significant grain growth observed. Solid-state dominant sintering produced less dense samples, as shown in Fig. 1.

An examination of the sintered samples (32 W_s_, 42 W_s_, 56 W_s_, 63 W_s_, 71 W_s_, 77 W_s_ and 90 W_s_ samples) through EDS area mapping revealed multiple phases in bright matrix (HEA and/or W-rich, detailed analysis is presented in following sections). The presence of a Ti-rich phase, segregated along the grain boundaries, in a HEA/W-rich matrix was also confirmed from the elemental area maps shown in the Supplementary Information (Figure S5). The samples with a high content of W contain V- and Cr-rich phases, whereas the Ti-rich molten phase in samples with low W content (<71at.%) prevented the formation of V- and Cr-rich phases and facilitated a solid solution with relatively large grain sizes^120, 137^.

**Figure 3:**
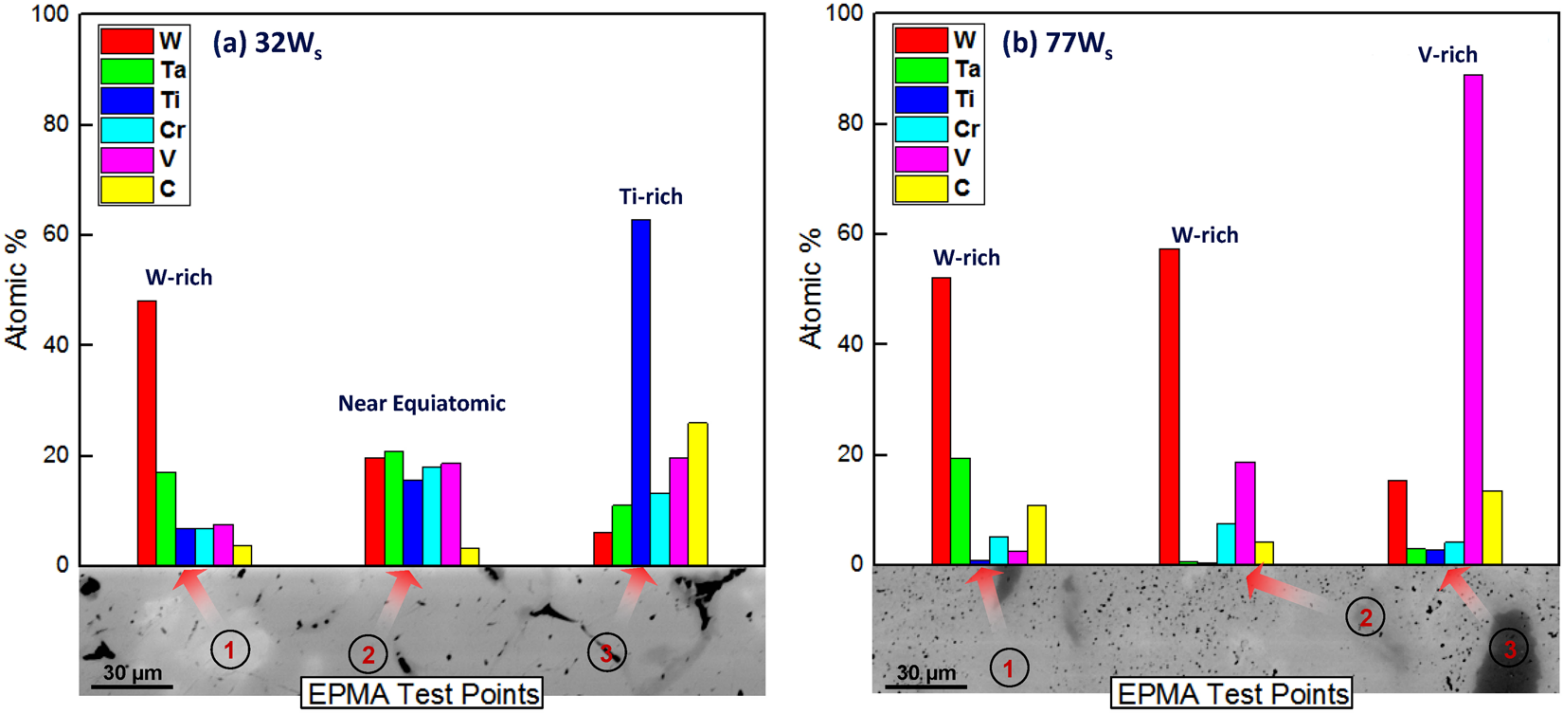
Various alloy compositions observed in (**a**) 32 W_s_ and (**b**) 77 W_s_.

An electron probe micro-analysis (EPMA) was carried out to determine the content of the constituent elements in the various phases (W-, Ti-, V- and Cr-rich) observed in the elemental mapping. The chemical composition determined by EPMA confirmed a large fraction of single phase HEA matrix containing other phases. The test was repeated at various points with the same sample. Figure 3 shows the typical results after varying the compositions of the 32 W_s_ and 77 W_s_ samples at different test points, indicating the development of three major phases in the bulk samples. The near-equiatomic nominal composition (32 W_s_) gives rise to the formation of a HEA matrix with a near-equiatomic composition (88 vol.%) with ~8 vol.% and ~4 vol.% W- and Ti-rich phases, respectively. In contrast, ~4 vol.% and ~5 vol.% Ti- and V-rich phases, respectively, were observed in the W-rich alloy matrix of the 77 W_s_ sample.

Attempts were made to increase the chemical homogeneity in the microstructure via annealing at 1200 °C, 1300 °C, 1400 °C and 1500 °C. The samples were held at these temperatures for up to one hour (six times longer than the sintering time). The temperature was increased at 10 °C/min, with a subsequent furnace cooling step. However, no significant change in the microstructure and hardness after the heat treatment was observed, as shown in Figure S1. This revealed the thermal stability of the phases (shown in Figs 1, 3 and S5) which had formed during the SPS process.

**Figure 4:**
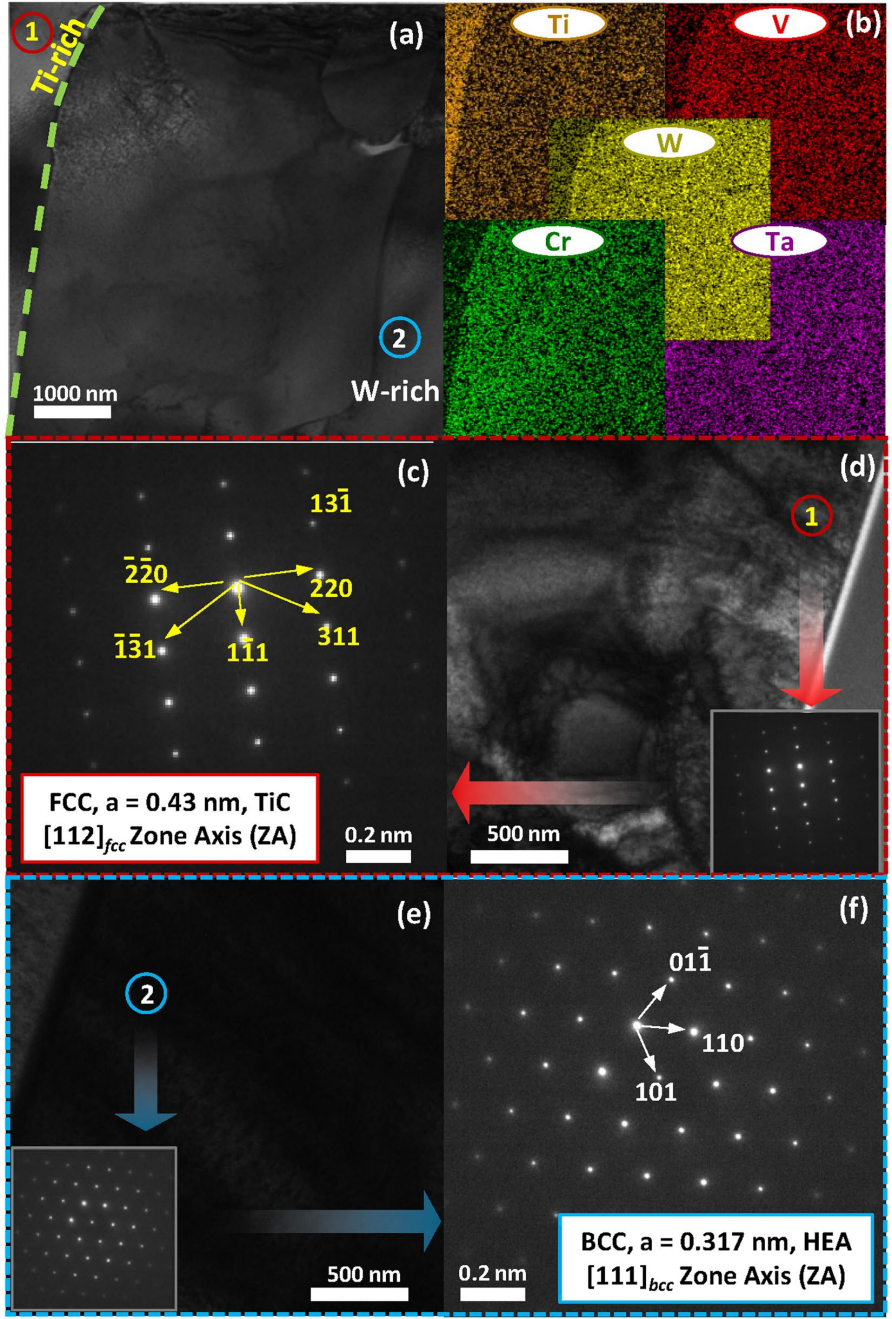
(**a**) TEM microstructure, (**b**) elemental mapping, (**c** and **d**) SADP of TiC and (**e** and **f**) SADP of the HEA phase as observed in 32 W_s_.

In order to investigate the chemical nature of the various regions (W-, Ti-, V- and Cr-rich), the samples were examined under a transmission electron microscope. Typical TEM analysis results of the 32 W_s_ sample are shown in Fig. 4.

The HEA- and Ti- rich regions in the 32 W_s_ (HEA) sample marked with numbers on the microstructures (Fig. 4a) with the help of elemental mapping are shown in Fig. 4(b). A selected-area electron diffraction (SAED) pattern analysis of the Ti-rich region, from the [112]_fcc_ zone axis (ZA), as shown in Fig. 4(c and d), respectively, exhibited fundamental FCC reflection with a lattice parameter of 0.43 nm (calculated). The Ti-rich region (Fig. 4b), FCC crystal structure (Fig. 4(c and d)), and lattice parameter of 0.43 nm (calculated) confirm the formation of TiC in the dark regions observed in the back-scattered electron (BSE) images of xW_s_. The formation of TiC can be explained by the diffusion of carbon in the samples from the graphite mold during the spark plasma sintering step^125–132^ (details are given in Section S4).

It has been reported that the formation of TiC can improve the physical and chemical properties of alloys^72^. The TiC, dispersed along the grain boundaries, restricts the migration of grain boundaries which results in grain boundaries strengthening^27^. The TEM microstructures also showed clean TiC/HEA interfaces without any precipitates, the good interface joint between TiC/HEA ensures the load transfer while loading^28^. The W-based composite having uniformly distributed TiC within the grains and along the grain boundaries can withstand heat flux as high as 200 MW/m^2^, which is nearly 100% higher than the flux sustained by pure W^29^. An increase in the amount of the evenly distributed TiC in the W matrix (inside the grains and along the boundaries) also increases the hardness creep strength and modulus of elasticity^27, 30, 73^. The presence of TiC in the grains and on the grain boundaries also favors fusion applications, as it increases the recrystallization temperature and irradiation resistance^29–31^. TiC does not have a significant effect on the deuterium retention behavior of W-based materials^74, 75^. The dispersion of carbides in the grain boundaries of W leads to grain boundary strengthening and provides some resistance to grain boundary migration at elevated temperatures^27^. Therefore, TiC is considered to be beneficial in high-temperature and plasma-facing alloys. The volume fraction of TiC in the xW_s_ samples determined by analyzing the SEM images was found to vary from 1.1 to 8.5% (Table S3). The volume fraction of the TiC content, as determined by analyzing the SEM images, exhibits an increasing trend from 90 W_s_ to 42 W_s_, which may account for the increase in the Ti content from 90 W_s_ to 42 W_s_. This observation suggests a means of controlling the TiC content in xW_s_ alloys. The irregular size of the TiC phase was observed to vary from 10 to 25 µm.

However, other means of reducing the formation of TiC (if required) are also available, such as diffusion barriers of alumina, platinum and tantalum^125^ between the graphite and the sample during the spark plasma sintering process.

The selected-area diffraction patterns (SADPs) of the HEA phases from [111]_bcc_ are shown in Fig. 4(e and f). The fundamental reflections of the BCC crystal lattice from the HEA region were observed (Fig. 4f), in good agreement with the XRD and SEM results. The lattice parameter of the BCC crystal structure lattice observed as a bright matrix in the BSE images (i.e., 0.317 nm (calculated)) is evidence of similarity HEA matrix with W-rich BCC alloy (as observed by XRD examination, shown in Figure S4). Similarly, the TEM analysis of TiC and W-rich phase (instead of HEA) observed in xW_s_ (x = 42 to 63, HEA derivatives), is represented in the Supplementary Information in Figure S7.

**Figure 5:**
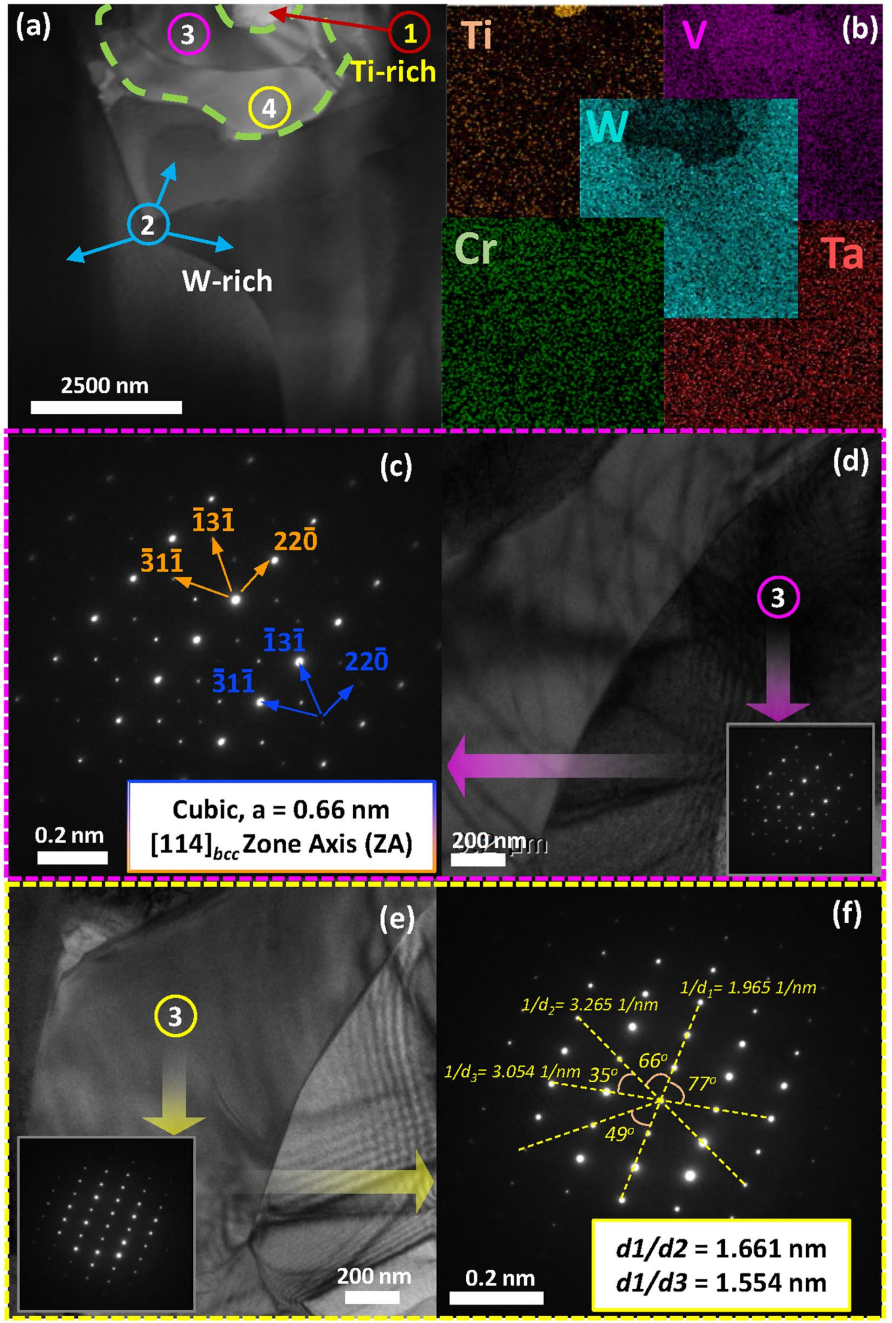
(**a**) TEM microstructure, (**b**) elemental mapping, (**c** and **d**) SADP of the C15 laves phase and (**e** and **f**) SADP of a complex crystal structure dissimilar to any of the fundamental crystal structures, as observed in 90 W_s_.

The 71 W_s_, 77 W_s_ and 90 W_s_ samples, which show V- and Cr-rich phases in the EDS elemental mapping results (Figs S5 and 3), were tested by TEM for a SADP analysis. Figure 5 shows the TEM analysis results of the 90 W_s_ sample as a typical xW_s_ sample with an x value ranging from 71 to 90. The Ti- and W-rich phases, as indicated by the numbers 1 and 2, respectively, in Fig. 5a, are the TiC and W-rich BCC solid solution, as explained by the SADP analysis results shown in Figs 4 and S7. In addition to these two phases, 90 W_s_ contains V- and Cr-rich regions as well, as shown in Figs S5, 3 and 5b. The grains having such phases are indicated by points 3 and 4 in Fig. 5a. The SADP from the [114]_bcc_ zone axis of the grains numbered as 3 shows the presence of a superlattice, i.e., two distinct crystals, which is a characteristic of the C15 cubic Laves phase^138^.

The C15 Laves phase has cubic symmetry^76^, a structure similar to that of MgCu_2_, and contains two sublattices^77^. A short passage on the formation of Laves and their effects on the properties along with the expected C15 cubic Laves phases in the W_x_TaTiVCr alloy system (Table S4) is given in the Section S5 of Supplementary Information.

The lattice parameter calculated by indexing the crystallographic reflections, obtained from the V-rich grain (numbered as 3), as shown in Fig. 5c, is 0.66 nm, which closely matches the lattice parameters of the binary C15 VV_2_^139^ and Ti(Cr,V)_2_^140^ Laves phases. The SADP of the adjacent vanadium-rich grain, numbered as 4 in Fig. 5a, shows a rather complex structure dissimilar to any of the fundamental cubic or hexagonal structures. The relatively low liquid phases during the sintering of xW_s_ when x = 71 to 90 may be related to the segregation and formation of Laves intermetallics in the samples with higher at.% levels of W^71, 72, 141^.

**Figure 6:**
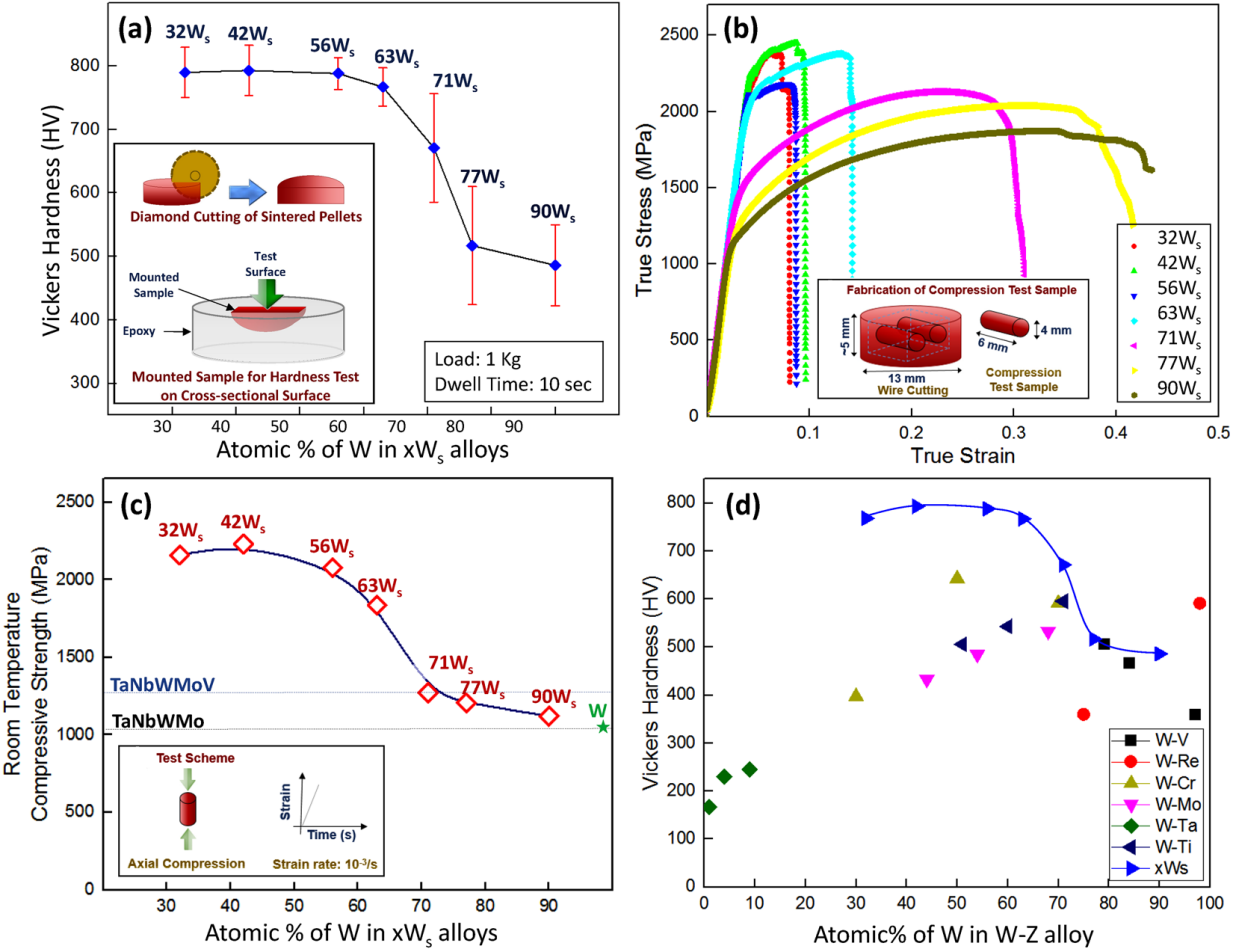
(**a**) Effects of the composition on the hardness of xW_s_, (**b**) true stress-true strain curves of xW_s_, (**c**) variation in the compressive yield strength and fracture strain of xW_s_ with varying compositions and a comparison with pure tungsten (this work), TaNbWMoV and TaNbWMo^93^ and (**d**) Comparison of the hardness levels of xW_s_ with W-V (W-3.5at.%V, W-16at.%V and W-21.3at.%V)^44^, W-Re (W-2at.%Re^48^ and W-24.7at.%Re-SPS at 1500 °C)^47^, W-Cr (W-30at.%Cr, W-50at.%Cr and W-70at.%Cr)^58^, W-Mo (W-32.2at.%Mo, W-45.5at.%Mo and W-56.3at.%Mo)^56^, W-Ta (W-99at.%Ta, W-96at.%Ta and W-90.7at.%Ta)^50^, and W-Ti (W-29at.%Ti, W-40.2at.%Ti and W-48.8at.%Ti)^54^.

The effects of the composition on the mechanical behavior of the xW_s_ HEA samples were assessed by subjecting the cross-section of the sintered samples to a micro-Vickers hardness test. At least ten readings were obtained in the HEA matrix to avoid the effect of chemical inhomogeneity in order to represent the actual hardness of xW_s_, and the average value was used. Figure 6(a) summarizes the influence of the chemical composition on the hardness of xW_s_.

Figure 6(a) indicates that the development of xW_s_ alloys (x = 32 to 90) by simple mixing can play a significant role in fulfilling the requirements of enhanced hardness. The increased hardness is attributed to differences in the atomic radii of the constituents of the high-entropy alloy (x = 32). Previous research^77, 88, 98, 133^ showed that differences in the atomic radii of the constituents hindered dislocation movement by producing a locally distorted and stressed crystal structure. Moreover, by changing the composition from 90 W_s_ to 42 W_s_, the hardness can be increased to ~790 Hv, which is much higher than the hardness of pure tungsten, i.e., ~350 Hv.

An analogy between the hardness and compressive yield strength trends can be seen by comparing Fig. 6(a) and (c). With a decrease in x from 90at.% to 32at.%, the compressive yield strength increased to ~2200 MPa (i.e., twice as high as that of pure tungsten) and almost double the strengths of TaNbWMoV and WNbWMb as well^93^. The fracture strain was found to increase, as shown in Fig. 6(b), with an increase in the x values in xW_s_. This improved fracture strain accounts for the higher W content, as pure W shows substantial plastic deformation while under compression at room temperature^142^, unlike its brittle behavior under tensile and impact loading conditions^142, 143^. The presence of TiC also accounts for the improved hardness and strength^144–146^ of xW_s_ owing to dispersion strengthening. The extent of the improvement of the mechanical behavior of xW_s_ can also be appreciated by comparing the hardness of xW_s_ with these levels of various W-based binary alloys, as shown in Fig. 6(d). In addition, the xW_s_ alloys present hardness levels higher than those of several W-based binary alloys^79, 85, 86, 95, 96, 101, 102, 147^.

Table 2, which summarizes the physical, mechanical and thermodynamic parameters of xW_s_ sintered at 1600 °C, provides insight into the enhanced hardness and strength of xW_s_.

**Table 2:** Summarized physical and mechanical behavior with the thermodynamic properties of xW_s_ sintered at 1600^o^ C.

Name (xW_s_)	Hardness (HV)	Yield Strength (MPa)	Measure Density (g/cm^3^)	Atomic Size Diff. (%)	Entropy of Mixing (J/K-mol)
90 W_s_	486	1206	16.5	1.74	3.31
77 W_s_	517	1208	16.5	2.47	6.15
71 W_s_	671	1473	15.7	2.71	6.89
63 W_s_	767	2187	14.9	3.00	7.96
56 W_s_	788	2144	14.5	3.21	8.58
42 W_s_	793	2314	13.6	3.53	9.70
32 W_s_	768	2265	13.4	3.76	10.38

The strength and hardness levels of other high-entropy alloys are attributed to dispersion strengthening^46^, nano-scale twins^118^, order-strengthening effects, grain boundary strengthening, and solid-solution strengthening^72^. However, the increase in the hardness, the room-temperature compressive yield strength, the relative density and the atomic size difference with an increase in the cumulative atomic fraction of Ta, Ti, Cr and V in the W matrix reveal the dominant role of solid-solution strengthening, which increases with an increase in the fraction of the constituent atoms and the atomic size difference^65^. In addition to the elastic interaction between the stress field of dislocations and atoms^65^, which accounts for nearly fifty percent of the strength of HEA^72, 98, 148^, the dynamic drag effect, which arises due to non-uniform stresses and causes acceleration and deceleration of dislocation sliding, leads to strong strengthening as well^98^.

The average grain sizes of the xW_s_ alloys as produced by the powder metallurgical process are ~30–50 µm (when x = 32 to 63at.%) and ~3 µm (when x = 71 to 90), as evident in Fig. 1 and as represented in Figure S8. The liquid-phase assisted sintering of xW_s_ with x values ranging from 32 to 63 produced larger grains than those produced by solid-state dominant sintering (such as xW_s_ with x = 71–90 at.%). Other refractory HEA types, such as WNbMoTa and WNbMoTaV, when produced via vacuum melting show a structure with corresponding grain sizes of 200 µm and 80 µm^89, 90^. The relatively small grain size also accounts for the improved hardness and strength.

This study presented the microstructures and room-temperature mechanical properties of a HEA (32 W_s_) with *in-situ* TiC prepared by elemental powder mixing followed by spark plasma sintering. The enhanced hardness and strength of the 32 W_s_ due to distorted lattice and solid solution strengthening were also revealed. Additionally, several W-based alloys were derived from HEA by gradually increasing x up to 90 at.% in xW_s_. The solid solution strengthening imparted enhanced hardness and strength in W-based alloys as well. The improved hardness and strength of xW_s_ emphasizes the potential of this facile method (i.e., elemental powder mixing) for the development of refractory HEAs. The chemical composition of xW_s_ fulfills the reduced-activation criteria (which are commonly followed for producing reduced-activation steels (Section S1))^105, 106, 113^. However, the analysis of reduced-activation behavior, irradiation resistance^149^, hydrogen retention^22^ and high temperature mechanical properties^90^ will be necessary prior to its utilization as an armor material in future fusion reactors including DEMOnstration power station (DEMO), where it can be applied as an armor material for ‘divertor’ and ‘first wall’ to face fusion plasma, neutron flux, tritium and plasma dust at a temperature level which has never been seen before.

## Conclusions

A powder metallurgical technique using elemental powder mixing has been exploited by developing and analyzing a novel high-entropy alloy (W_x_TaTiVCr, simplified as xW_s_) and its derivative alloys for the development of reduced-activation alloys for future fusion power plants. Various compositions realized by varying the W content from 32 to 90at.% with *in-situ* TiC were prepared by consolidating a powder mixture through spark plasma sintering at 1600 °C. The relative density of the alloys sintered at 1600 °C was found to increase with a decrease in the W content. The characterization of xW_s_ samples by XRD showed the BCC crystal structure of the alloys. A microstructural examination by SEM-EDS revealed multiple phases in the microstructures as bright, gray and dark regions. Moreover, various W-rich phases were observed in the matrix of the sintered samples. An investigation of these phases under TEM and a selected-area diffraction pattern analysis disclosed the presence of TiC in the W-rich matrix, as observed in BSE images. Moreover, C15 Laves phases were found in V- and Cr-rich regions when the W content exceeded 70at.%. The increasing lattice strain and solid-solution strengthening produced xW_s_ (when x = 32 to 63at.%) with hardness and strength levels which are twofold higher than those of pure tungsten and previously reported W-containing refractory HEAs. This study revealed the potential role of high-entropy alloys with enhanced physical and mechanical characteristics for forthcoming fusion power reactors.

### Data availability statement

The datasets generated during and/or analysed during the current study are available from the corresponding author on reasonable request.

## Electronic supplementary material

Supplementary information
