# Supplementary material for: Powder Metallurgy Processing of a WxTaTiVCr High-Entropy Alloy and Its Derivative Alloys for Fusion Material Applications
Source: Sci Rep. 2017 May 16;7:1926. doi: 10.1038/s41598-017-02168-3 (PMC5434025; doi:10.1038/s41598-017-02168-3)
Supplement: Supplementary file 1 — Supplementary information [file 41598_2017_2168_MOESM1_ESM.pdf]

Supplementary Information

Powder Metallurgy Processing of a W_xTaTiVCr High-Entropy Alloy and Its Derivative Alloys for Fusion Material Applications

Owais Ahmed Waseem, Ho Jin Ryu*

Department of Nuclear and Quantum Engineering, Korea Advanced Institute of Science and Technology, 291 Daehakro, Yuseong-gu, Daejeon 34141, Republic of Korea

*Co-corresponding Author: Tel.: +82–42–350–3812, Fax: +82–42–350–3810,

E-mail address: hojinryu@kaist.ac.kr (Ho Jin Ryu)

S1. W_xTaTiVCr for the Development of Reduced-Activation Alloys

The fusion plasma applications don't only exert harsh environment on the reactor materials, but it also expect the materials to show minimum or no activation[1], so that the reactor materials can be accessed for maintenance and recycling during service and at the end of their service lifetime[1], respectively. The management of radioactive waste of plasma facing materials is an important consideration[1] which will include transportation, disposal and/or recycling of the decommissioned materials[2]. The whole waste management scenario depends upon the activation characteristics of the materials[2]. Based upon the limit of the dose rate (25 µSv/h) of the materials employed in fusion plasma facing applications, the use of Mo, Nb, Ni, Co, Al, Ag[3], Cu, N, Ho, Bi, Sm, Lu, Dy, Gd and Cd[1] is restricted to extremely low levels. Complying with these restrictions, the refractory W_xTaTiVCr system assures the reduced activation properties.

In addition to reduced activation properties, irradiation resistance of nuclear materials is also essential for the good performance of nuclear reactor materials[4]. The radiations

stimulate displacement of atoms which generates vacancy and interstitial sites[5], which evolves towards cascade collision, recombination (of vacancy and interstitial) and absorption in dislocations and grain boundaries[6]. A popular way to reduce the irradiation induced defects is to generate high density of defect sinks[7].

Lu et al. explained inherent irradiation resistance of HEA by relating this with enhanced scattering of displaced atoms due to lattice distortion[7] which results in limited linear mobility of displaced atoms[7], and the atoms are confined to vacancy concentrated area, as a result the probability of recombination increases, consequently, the density of defects in irradiated HEA reduces[7]. These kinetic constraints in the diffusion of atoms also decreases the irradiation-induced precipitation in HEA and promote stable structure[4]. Because of the difference in atomic sizes, the HEA lattice is more stressed at atomic level as compare to other alloys.

The successful nuclear applications of tungsten[8], good performance of Ti and Cr in fusion neutron spectrum[9], high irradiation resistance of Ta[10] and V[11], no long term activation in any of the constituent[1,3,9] in conjunction with phase stability (as the annealing of sintered samples at 1200, 1300, 1400 and 1500°C up to 1 hour didn't cause any significant change in microstructure and hardness, as illustrated in Figure S1) and self-healing ability of HEA anticipate the improved irradiation resistance of $W_xTaTiVCr$ system and make it a potential candidate for fusion and plasma facing material.

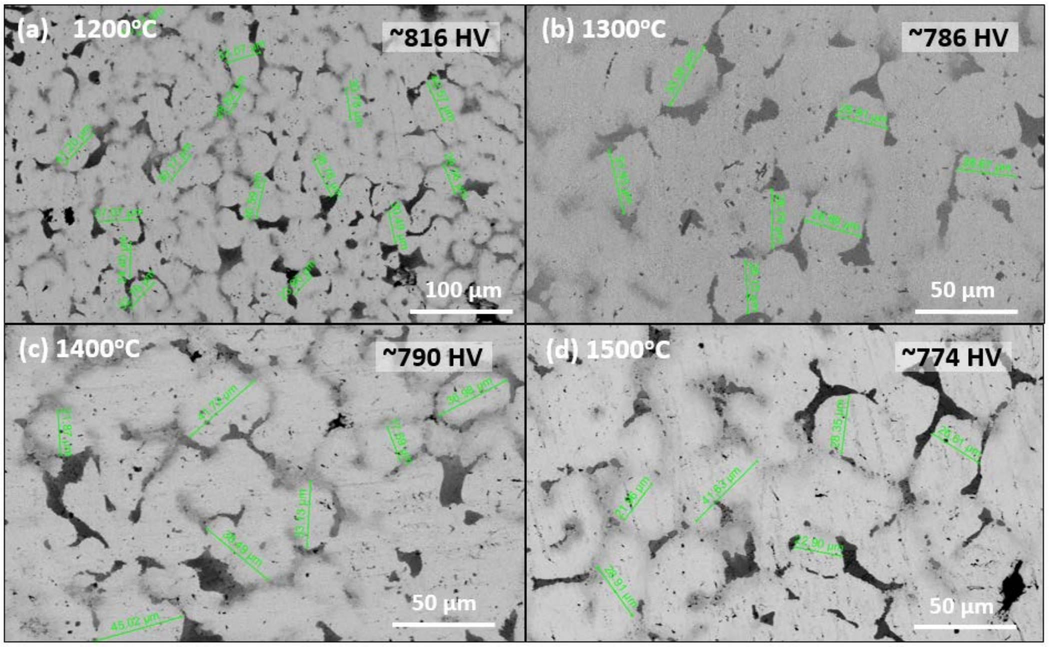

Figure S1. *Representative SEM microstructures of 56Ws and Hardness annealed at (a) 1200°C (b) 1300°C (c) 1400°C and (d) 1500°C up to 01 hour*

Several irradiation resistant refractory high entropy alloys have been reported, however, the published research lacks in HEA having refractory nature and reduced activation properties. A comparison of W_xTaTiVCr alloy, given in Table S1, with already reported HEAs emphasized upon the novel composition of W_xTaTiVCr complying with reduced-activation criteria.

Table S1

Comparison of HEA (32W_S) with other refractory HEAs.

HEA	Reduced activation Properties	Strength (MPa)	Reference
$W_{0.32}Ta_{0.18}Ti_{0.18}V_{0.20}Cr_{0.19}$ (32W_S)	Yes	2265	This Work
$V_{20}Nb_{20}Mo_{20}Ta_{20}W_{20}$	No	1040	[12]

$Nb_{25}Mo_{25}Ta_{25}W_{25}$	No	1000	[12]
NbCrMoTaTiZr	No	1595	[12]
TaNbHfZrTi	No	960	[12]
TaNbWMo	No	1050	[12]
TaNbWMoV	No	1250	[12]
TiZrHfNbTa	No	890	[13]
TiZrNbV	No	1104	[14]
$TiZrNbVMo_x$ (x=0.3-2)	No	1289-1765	[14]
$TiZrNbV_{0.3}$	No	866	[14]
$TiZrNbV_{0.3}Mo_x$ (x=0.1-1.5)	No	932-1576	[14]
$Nb_{25}Mo_{25}Ta_{25}W_{25}$	No	1058	[15]
$V_{20}Nb_{20}Mo_{20}Ta_{20}W_{20}$	No	1246	[15]
$Ti_{20}Zr_{20}Hf_{20}Nb_{20}V_{20}$	No	1170	[16]
$Ti_{20}Zr_{20}Hf_{20}Nb_{20}Cr_{20}$	No	1375	[16]
HfNbTaTiZr	No	1250	[17]
TiZrHfNbTa	No	910	[18]
$Ti_{20}Hf_{20}Zr_{20}Ta_{20}Nb_{20}$	No	980	[19]
$Mo_{0.5}NbHf_{0.5}ZrTiC_{0.1}$	No	1183	[20]
$Mo_{0.5}NbHf_{0.5}ZrTiC_{0.3}$	No	1201	[20]
$Mo_{0.5}NbHf_{0.5}ZrTi$	No	1176	[20]
MoNbHfZrTi	No	1719	[21]
HfNbTaTiZr	No	929	21
$HfMo_xNbTaTiZr$ (x=0-1)	No	1015-1512	[21]
$Al_xCoCrFeNi$ (x=0.1-0.3)	No	250-220	[4,22]

S2. Optimization of sintering

Due to high melting points, the sintering of refractory metals and alloys is relatively difficult. In order to find out optimized conditions for improved diffusion and enhanced sintering, a series of experiments were carried out and effect of sintering temperature on promoting diffusion and enhancing sintering were studied.

The role of thermal diffusion in achieving improved sinterability of our HEA sample $W_{0.3}(TaTiVCr)_{0.7}$ was analyzed by carrying out SPS at 1300°C, 1400°C, 1500°C and 1600°C up to 10 minutes. The prominent effect of sintering temperature on enhanced diffusion was observed, as shown in Figure S2. The gradual homogenization of microstructure and increase in relative density was observed with increasing temperature. The sample sintered at 1600°C achieved >99% density with homogeneous microstructure. Hence, 1600°C and 10 minutes were employed for SPS of further W_xTaTiVCr samples.

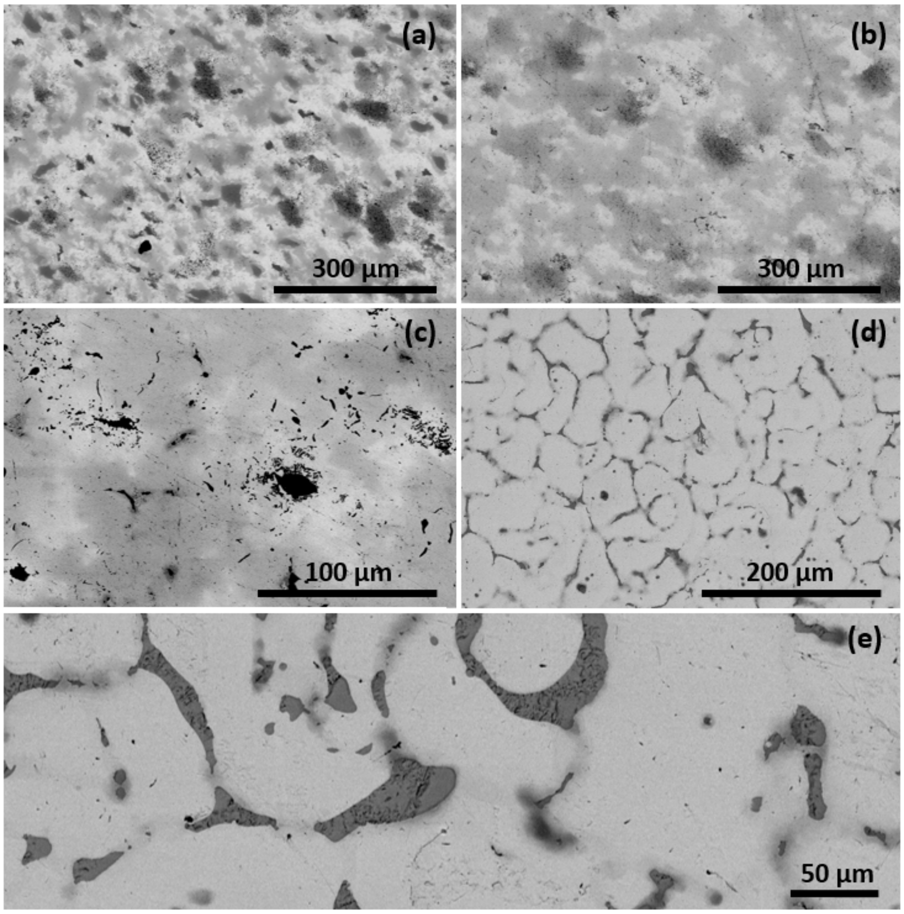

Figure S2. *Representative SEM microstructures of $W_{0.3}(TaTiVCr)_{0.7}$ sintered at (a) 1300°C (b) 1400°C (c) 1500°C and (d & e) 1600°C up to 10 minutes*

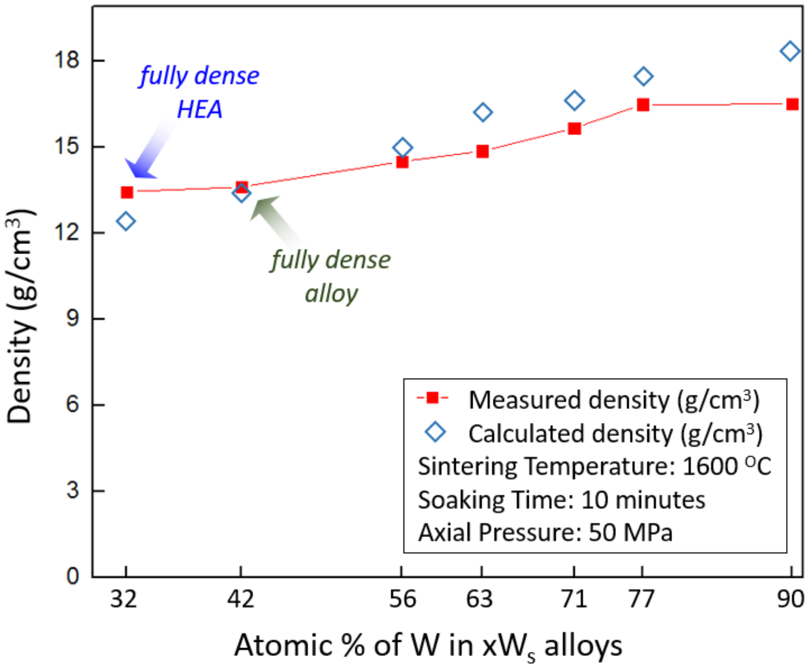

Figure S3. *The variation in the density of the xW_s alloys with varying compositions.*

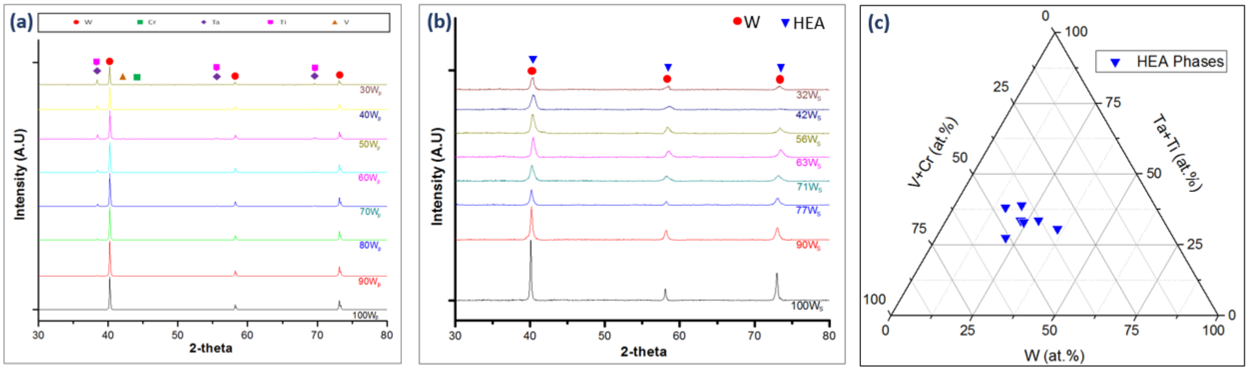

Figure S4. *XRD patterns of the (a) powder mixture xW_p and (b) sintered samples xW_s and (c) Compositions of HEA phases in xW_s (determined by EPMA)*

S3. Sintering mechanism of $W_xTaTiCrV$ alloy system

In addition to the higher configuration entropy of xW_s, the valence electron concentration (VEC) of $xW_s \leq 6.87$[23], and the negligible mixing enthalpy, which spontaneously led to the development of solid solution[24-25], the liquid phase sintering (LPS) played a major role in sintering of xWs samples at 1600°C, similar to the sintering of AlCoCrFeNi[25], W-Ni-X powder (X = Fe, Cu, Cr, or Co)[26] and WC–Co where liquid phase formed due to melting of low melting point constituent and enhanced sintering of powders[27]. A small fraction of liquid phase may cause drastic improvement in sintering[28] due to fast diffusion and the dissolution of solid particles[29]. In xWs alloy systems, certain inhomogeneity which cannot be perfectly removed in simple mixing of powder, developed highly localized Ti-Cr and Ti-Cr-V based alloys having a low melting point in the range of sintering temperature (subject to the ratio of the constituents)[30], as illustrated in Fig. S7. The liquid phase containing Ti, Cr and V seeped between the solid particles and causes wetting due to the mutual solubility of constituents among each other. Thus the liquid phase, which not only promoted re-arrangement of particles, but also improved diffusion of atoms[27] resulted in rapid sintering of xWs. The SEM microstructures and decrease in densities (Fig. 1 and Fig. S3, respectively) with increasing W content in xW_s also supports the role of Ti-Cr and Ti-Cr-V based liquid phase in rapid sintering. Due to a low fraction of liquid phase, reduction in grain size and density with increasing W content was observed.

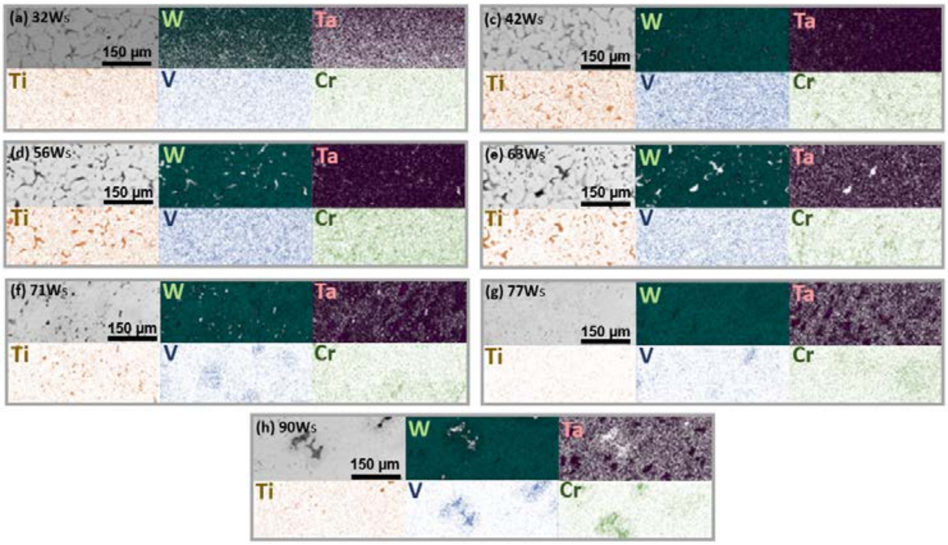

Figure S5. *EDS area mapping of the sintering of xW_s at 1600^oC.*

S4. Formation of TiC in W_xTaTiVCr alloy system

Koc et al. reported the synthesis of TiC occurs between 1200^oC and 1600^oC[31] through direct carbonization of Ti (Ti + C → TiC)[32]. The thermodynamics of TiC supports its in-situ formation[32]. The comparison of free energies of the carbides of constituents, as reported by Shatynski[33], reveals that the most negative heat of formation (i.e., -44.1 kcal/mol) of TiC in the sintering temperature range as compared to that of the carbides of other constituent elements (W, Ta, V[33-34] and Cr[35])[32] results in the presence of TiC in sintered samples. The heats of formation of constituent elements are given in table S1[34].

Table S2

Heats of formations of expected carbides in W_xTaTiVCr system[34].

Compound	-ΔHf (kcal/mole)
TiC	44.1
V_2C	16.5
VC	24.5
Ta_2C	24.9
TaC	34.1

Cr_3C_2	5.5
W_2C	6.3
WC	9.7

The carbon taken up from the graphite mold reacts with Ti to form TiC[31,36-40]. The microstructural analysis revealed an 80-µm-thick TiC-rich surface layer, whereas the rest of the sample showed evenly distributed TiC (as shown in Fig. S6).

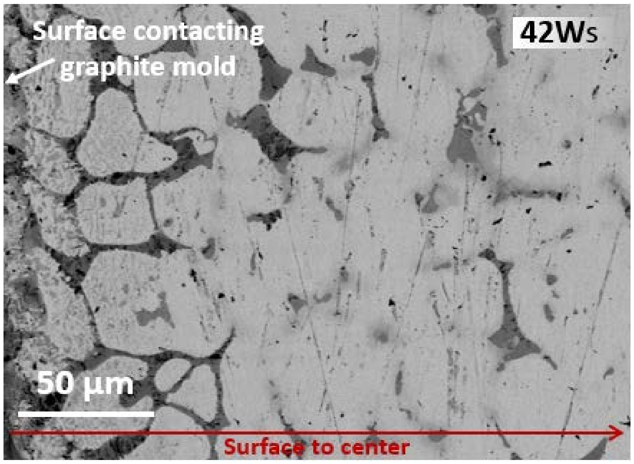

Figure S6. The distribution of TiC from surface to center.

Due to the attractive properties including high melting point, high thermal shock resistance, and good physical and chemical sputtering resistance[41], TiC is added in several alloys as a reinforcement[32].

Table S3

Calculated weight fractions of TiC in xW_s.

Name (xW_s)	Real Samples		Volume fraction of TiC (%)
$32W_s$	$W_{0.32}Ta_{0.18}Ti_{0.18}V_{0.20}Cr_{0.19}$	HEA	2.24
$42W_s$	$W_{0.42}Ta_{0.15}Ti_{0.14}V_{0.14}Cr_{0.14}$	HEA derivatives	8.50
$56W_s$	$W_{0.56}Ta_{0.15}Ti_{0.09}V_{0.11}Cr_{0.09}$		6.05
$63W_s$	$W_{0.63}Ta_{0.09}Ti_{0.09}V_{0.09}Cr_{0.09}$		6.20
$71W_s$	$W_{0.71}Ta_{0.04}Ti_{0.07}V_{0.07}Cr_{0.07}$		3.30
$77W_s$	$W_{0.77}Ta_{0.05}Ti_{0.07}V_{0.05}Cr_{0.06}$		2.50

$90W_s$	$W_{0.90}Ta_{0.03}Ti_{0.02}V_{0.03}Cr_{0.02}$		1.10

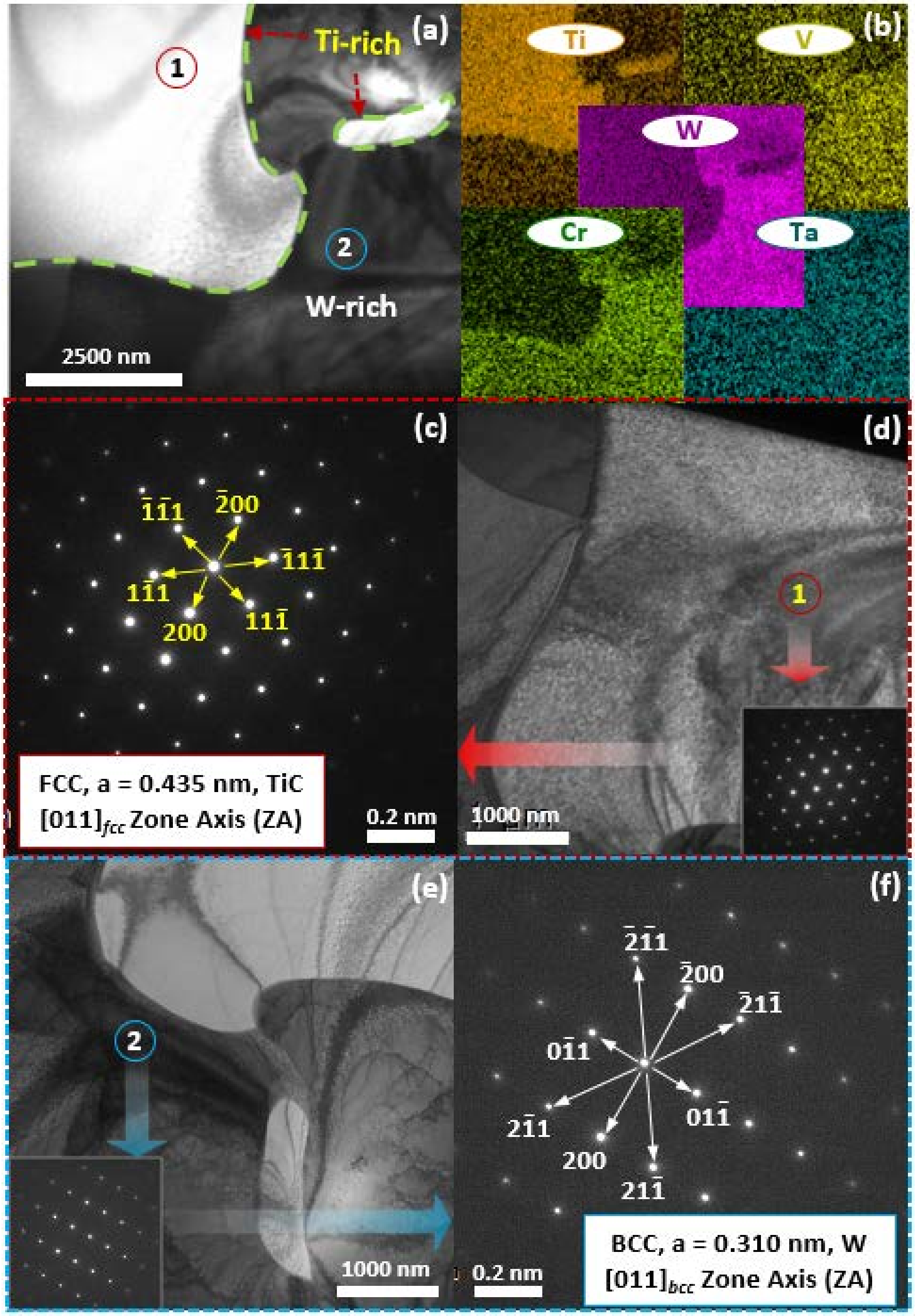

Figure S7. *(a) TEM microstructure, (b) elemental mapping, (c & d) SADP of TiC and (e & f) SADP of the W-rich phase as observed in $56W_S$.*

S5. Laves phases

The transition metallic elements having highly negative heat of mixing can form the intermetallic Laves[42] having non-metallic bonding (i.e. ionic or covalent bonding). More than one type of bond can also be present in Laves intermetallics[42]. The atomic architecture of Laves phases is represented as AB_2, where A is the element having high electropositivity and a large Gold-schmidt radius[43-44]. In C15 Laves phase, A is surrounded by 12 B atoms and B is surrounded by 6 atoms of each element[44]. The lattice parameter of Laves phases may experience slight variation with the varying temperature[45]. The relatively low liquid phases during the sintering of xW_s when x=71 to 90 may be related to the segregation and formation of Laves intermetallics in the samples with higher at.% levels of W[46-48]. The expected Laves phases in W_xTaTiVCr system are giving in Table S2

Table S4

Possible C15 (cubic) Laves phases in the W_xTaTiVCr alloy system.

**Laves Phases C15 (cubic)**	**Lattice Parameter a (nm)**
TaV_2	0.715[43,49], 0.713[50], 0.722[50-51], 0.7118[50], 0.712[50-51], 0.71597[45], 0.7163[45], 0.714[45], 0.709[49]
$TiCr_2$	0.6944[52], 0.6857[53], 0.69[54]
VV_2	0.688[49]
VTa_2	0.753[49]
$TaTa_2$	0.764[49], 0.769[55]
TiV_2	0.712[51]
$CrCr_2$	0.658[55]
$TaCr_2$	0.697[56] 0.691[55], 0.700[55]
$CrTa_2$	0.748[55]
$Ti(Cr,V)_2$	0.694, 0.695, 0.696[57]
$(Ti,V)(V,Cr)_2$	Lattice parameter is not given[57]

The high temperature properties of Laves phases are attractive[52] such as good oxidation resistance[58] but they are very brittle at room temperature[14,52]. The even

distribution of fine Laves particles in the grain or along grain boundaries are known to impart strengthening in the materials[59]. The improvement in creep resistance[60-61] and yield strength by forming Laves phases has been reported[61]. However, the non-uniform occurrence of Laves in larger size with increased inter-particle spacing[59] is not favorable as it can drastically reduce mechanical properties including strength, impact resistance and fracture toughness[14,62-63]. Acting as the sites for dislocation pinning and crack initiation, the Laves phases reduces fatigue limit as well[62]. A reduction in creep resistance due to Laves phases has also been reported[62]. The localized melting minimized the content of Laves HEAs and its derivatives. The decreasing amount of Ti, V and Cr in xW_s with increasing W content, reduces localized melting, therefore the $71W_{s,}$ $77W_s$ and $90W_s$ showed laves phases. However, the effects of Laves in xW_s is expected to be insignificant as their content was lower even than the detectable limit of XRD.

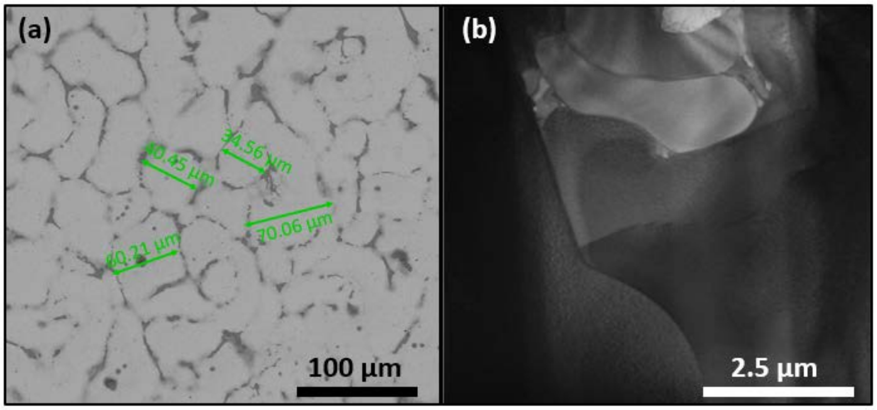

Figure S8. *BSE image of (a) 32W_S, and TEM image of (b) 90W_S showing the grain size.*

Table S2

Calculated weight fractions of TiC in xW_S.

Name (xW_S)	Real Samples		Volume fraction of TiC (%)
32W_s	$W_{0.32}Ta_{0.18}Ti_{0.18}V_{0.20}Cr_{0.19}$	HEA	2.24
42W_s	$W_{0.42}Ta_{0.15}Ti_{0.14}V_{0.14}Cr_{0.14}$		8.50
56W_s	$W_{0.56}Ta_{0.15}Ti_{0.09}V_{0.11}Cr_{0.09}$		6.05
63W_s	$W_{0.63}Ta_{0.09}Ti_{0.09}V_{0.09}Cr_{0.09}$	HEA derivatives	6.20
71W_s	$W_{0.71}Ta_{0.04}Ti_{0.07}V_{0.07}Cr_{0.07}$		3.30
77W_s	$W_{0.77}Ta_{0.05}Ti_{0.07}V_{0.05}Cr_{0.06}$		2.50
90W_s	$W_{0.90}Ta_{0.03}Ti_{0.02}V_{0.03}Cr_{0.02}$		1.10

References

1. Klueh, R. L., Cheng, E. T., Grossbeck, M. L. & Bloom, E. E. Impurity effects on reduced-activation ferritic steels developed for fusion applications. *J. Nucl. Mater.* **280,** 353–359 (2000).

2. Cheng, E. . Concentration limits of natural elements in low activation fusion materials. *J. Nucl. Mater.* **258–263,** 1767–1772 (1998).

3. Tan, L., Snead, L. L. & Katoh, Y. Development of new generation reduced activation ferritic-martensitic steels for advanced fusion reactors. *J. Nucl. Mater.* **478,** 42–49 (2016).

4. Yang, T. *et al.* Precipitation behavior of AlxCoCrFeNi high entropy alloys under ion irradiation. *Sci. Rep.* **6,** 32146 (2016).

5. Kumar, N. A. P. K., Li, C., Leonard, K. J., Bei, H. & Zinkle, S. J. Microstructural stability and mechanical behavior of FeNiMnCr high entropy alloy under ion irradiation. *Acta Mater.* **113,** 230–244 (2016).

6. Lu, C. *et al.* Direct Observation of Defect Range and Evolution in Ion-Irradiated Single Crystalline Ni and Ni Binary Alloys. *Sci. Rep.* **6,** 19994 (2016).

7. Lu, C. *et al.* Enhancing radiation tolerance by controlling defect mobility and migration pathways in multicomponent single phase alloys. *Nat. Commun.* **In press,** 1–8 (2016).

8. Abernethy, R. G. Predicting the performance of tungsten in a fusion environment: a literature review. *Mater. Sci. Technol.* **836,** 1–12 (2016).

9. Naujoks, D. *et al.* Tungsten as target material in fusion devices. *Nucl. Fusion* **36,** 671–687 (1996).

10. Dias, M. *et al.* Synergistic helium and deuterium blistering in tungsten–tantalum composites. *J. Nucl. Mater.* **442,** 69–74 (2013).

11. Smith, D. L., Chung, H. M., Loomis, B. A. & Tsai, H. C. Reference vanadium alloy V-4Cr-4Ti for fusion application. *J. Nucl. Mater.* **233–237,** 356–363 (1996).

12. Zhang, Y. *et al.* Microstructures and properties of high-entropy alloys. *Prog. Mater. Sci.* **61,** 1–93 (2014).

13. Dirras, G. *et al.* Microstructural investigation of plastically deformed $Ti_{20}Zr_{20}Hf_{20}Nb_{20}Ta_{20}$ high entropy alloy by X-ray diffraction and transmission electron microscopy. *Mater. Charact.* **108,** 1–7 (2015).

14. Wu, Y. D. *et al.* Phase composition and solid solution strengthening effect in TiZrNbMoV high-entropy alloys. Mater. Des. **83,** 651–660 (2015).

15. Senkov, O. N., Wilks, G. B., Scott, J. M. & Miracle, D. B. Mechanical properties of $Nb_{25}Mo_{25}Ta_{25}W_{25}$ and $V_{20}Nb_{20}Mo_{20}Ta_{20}W_{20}$ refractory high entropy alloys. *Intermetallics* **19,** 698–706 (2011).

16. Fazakas, É. *et al.* Experimental and theoretical study of $Ti_{20}Zr_{20}Hf_{20}Nb_{20}X_{20}$ (X=V or Cr) refractory high-entropy alloys. *Int. J. Refract. Met. Hard Mater.* **47,** 131–138 (2014).

17. Senkov, O. N. & Woodward, C. F. Microstructure and properties of a refractory $NbCrMo_{0.5}Ta_{0.5}TiZr$ alloy. *Mater. Sci. Eng. A* **529,** 311–320 (2011).

18. Kim, J. K., Sandlöbes, S. & Raabe, D. On the room temperature deformation mechanisms of a Mg-Y-Zn alloy with long-period-stacking-ordered structures. *Acta Mater.* **82,** 414–423 (2015).

19. Dirras, G. *et al.* Mechanical behavior and microstructure of $Ti_{20}Hf_{20}Zr_{20}Ta_{20}Nb_{20}$ high-entropy alloy loaded under quasi-static and dynamic compression conditions. *Mater. Charact.* **111,** 106–113 (2016).

20. Guo, N. N. *et al.* Microstructure and mechanical properties of in-situ MC-carbide particulates-reinforced refractory high-entropy Mo0.5NbHf0.5ZrTi matrix alloy composite. *Intermetallics* **69,** 74–77 (2016).

21. Juan, C. C. *et al.* Solution strengthening of ductile refractory HfMoxNbTaTiZr high-entropy alloys. *Mater. Lett.* **175,** 284–287 (2016).

22. Li, D. & Zhang, Y. The ultrahigh charpy impact toughness of forged $Al_xCoCrFeNi$ high entropy alloys at room and cryogenic temperatures. *Intermetallics* **70,** 24–28

(2016).

23. Pradeep, K. G. *et al.* Atomic-scale compositional characterization of a nanocrystalline AlCrCuFeNiZn high-entropy alloy using atom probe tomography. *Acta Mater.* **61,** 4696–4706 (2013).

24. Singh, S., Wanderka, N., Murty, B. S., Glatzel, U. & Banhart, J. Decomposition in multi-component AlCoCrCuFeNi high-entropy alloy. *Acta Mater.* **59,** 182–190 (2011).

25. Zhang, A. *et al.* Rapid preparation of AlCoCrFeNi high entropy alloy by spark plasma sintering from elemental powder mixture. *Mater. Lett.* **27,** 289–294 (2016).

26. Muddle, B. C. Interphase boundary precipitation in liquid phase sintered W-Ni-Fe and W-Ni-Cu alloys. *Metall. Trans. A* **15,** 1089–1098 (1984).

27. German, R. M., Suri, P. & Park, S. J. Review: Liquid phase sintering. *J. Mater. Sci.* **44,** 1–39 (2009).

28. Heady, R. B. & Cahn, J. W. An analysis of the capillary forces in liquid-phase sintering of spherical particles. *Metall. Trans.* **1,** 185–189 (1970).

29. Kingery, W. D. Densification during sintering in the presence of a liquid phase. I. Theory. *J. Appl. Phys.* **30,** 301–306 (1959).

30. Lee, J. Y., Kim, J. H. & Lee, H. M. Effect of Mo and Nb on the phase equilibrium of the Ti-Cr-V ternary system in the non-burning β-Ti alloy region. *J. Alloys Compd.* **297,** 231–239 (2000).

31. R. Koc, Lakewood, C. Gregory, Glatzmaier, Boulder, Process for synthesizing titanium carbide, titanium nitride and titanium carbonitride, United States Papent, Patent Number: 5,417,952, (1995). Available at: http://www.nrel.gov/docs/patents/16551.pdf (Access date: 04-01-2017)

32. Sahoo, P. & Koczak, M. J. Analysis of in situ formation of titanium carbide in aluminum alloys. *Mater. Sci. Eng. A* **144,** 37–44 (1991).

33. Shatynski, S. R. The thermochemistry of transition metal carbides. *Oxid. Met.* **13,** 105–118 (1979).

34. Storms, E. K., The Refractory Carbides, Refractory Materials (ed. Margrave, J. L.) 225-246 (Elsevier, 2016).

35. M. W. Chase, J. L. Churnutt, H. Prophet, R. A. McDonald, A. N. Syuerud, JANAF thermochemical tables, 1975 supplement, J. Phys. Chem. **4**, 1-172 (1975).

36. Khatri, S. & Koczak, M. Formation of TiC in in situ processed composites via solid-gas, solid-liquid and liquid-gas reaction in molten AlTi. *Mater. Sci. Eng. A* **162,** 153–162 (1993).

37. Choi, Y. & Rhee, S. W. Effect of aluminium addition on the combustion reaction of

titanium and carbon to form TiC. *J. Mater. Sci.* **28,** 6669–6675 (1993).

38. Mahmoodian, R., Hassan, M. a., Ghadirian, S. & Hamdi, M. Study of Ti+C Combustion Synthesis Reaction in a Controlled Declining Temperature State. *Combust. Sci. Technol.* **186,** 737–746 (2014).

39. Mahmoodian, R., Hassan, M. A., Hamdi, M., Yahya, R. & Rahbari, R. G. In situ TiC-Fe-Al_2O_3-TiAl/Ti_3Al composite coating processing using centrifugal assisted combustion synthesis. *Compos. Part B Eng.* **59,** 279–284 (2014).

40. Mahmoodian, R., Hassan, M. A., Rahbari, R. G., Yahya, R. & Hamdi, M. A novel fabrication method for TiC-Al_2O_3-Fe functional material under centrifugal acceleration. *Compos. Part B Eng.* **50,** 187–192 (2013).

41. Yin, X., Gotman, I., Klinger, L. & Gutmanas, E. Y. Formation of titanium carbide on graphite via powder immersion reaction assisted coating. *Mater. Sci. Eng. A* **396,** 107–114 (2005).

42. Zhu, J. H., Liu, C. T., Pike, L. M. & Liaw, P. K. Enthalpies of formation of binary Laves phases. *Intermetallics* **10,** 579–595 (2002).

43. Gunnæs, A. E., Karlsen, O. B., Olsen, A. & Zagierski, P. T. Phase relations and crystal structures in the Ag-Ga system. *J. Alloys Compd.* **297,** 144–155 (2000).

44. M, S. & Turchi, P. E. a. Phase stability in Ti-Cr and TI-V alloys: A theoretical investigation. *Physical Review B* **43,** 251–266 (1991).

45. Danon, C. A. & Servant, C. A thermodynamic evaluation of the Ta-V system. *J. Alloys Compd.* **366,** 191–200 (2004).

46. Toda-Caraballo, I. & Rivera-Díaz-Del-Castillo, P. E. J. Modelling solid solution hardening in high entropy alloys. *Acta Mater.* **85,** 14–23 (2015).

47. Del Grosso, M. F., Bozzolo, G. & Mosca, H. O. Modeling of high entropy alloys of refractory elements. *Phys. B Condens. Matter* **407,** 3285–3287 (2012).

48. King, D. J. M., Middleburgh, S. C., McGregor, A. G. & Cortie, M. B. Predicting the formation and stability of single phase high-entropy alloys. *Acta Mater.* **104,** 172–179 (2016).

49. Pavlů, J., Vřešťál, J., Chen, X. Q. & Rogl, P. Thermodynamic modeling of Laves phases in the TaV system: Reassessment using first-principles results. *Calphad Comput. Coupling Phase Diagrams Thermochem.* **35,** 103–108 (2011).

50. Charifi, Z., Reshak, A. H. & Baaziz, H. Electronic band structures of AV_2 (A = Ta, Ti, Hf and Nb) Laves phase compounds. *J. Phys. Condens. Matter* **21,** 25502 (2009).

51. Zhang, C. wen. Electronic structure and bonding properties for Laves-phase RV2 (R=Ti, Nb, Hf, and Ta) compounds. *Physica B: Condensed Matter* **403,** 2088–

2092 (2008).

52. Chen, K. C., Allen, S. M. & Livingston, J. D. Microstructures of two-phase Ti-Cr alloys containing the $TiCr_2$ Laves phase intermetallic. J. Mater. Res **12,** 1472-1480 (1997).

53. Chen, X., Wolf, W., Podloucky, R. & Rogl, P. Ab initio study of ground state properties of the Laves phase compounds $TiCr_2$, $ZrCr_2$, and $HfCr_2$. Phy. Rev. B **71**, 174101 (2005).

54. Ghosh, C., Basu, J., Ramachandran, D. & Mohandas, E. Alloy design and microstructural evolution in V-Ti-Cr alloys. *Mater. Charact.* **106,** 292–301 (2015).

55. Pavlů, J., Vřešťál, J. & Šob, M. Re-modeling of Laves phases in the Cr-Nb and Cr-Ta systems using first-principles results. *Calphad Comput. Coupling Phase Diagrams Thermochem.* **33,** 179–186 (2009).

56. Kuo, K. Ternary laves and sigma-phases of transition metlas. Acta Metall. **1**, 720-724 (1953).

57. Chen, K., Allen, S. & Livingston, J. Factors Affecting the Room-Temperature Mechanical Properties of $TiCr_2$-Base Laves Phase Alloys. *Mater. Sci. Eng. A* **242,** 162–173 (1998).

58. Zhao, J. C., Jackson, M. R. & Peluso, L. a. Determination of the Nb-Cr-Si phase diagram using diffusion multiples. *Acta Mater.* **51,** 6395–6405 (2003).

59. Kuhn, B. *et al.* Effect of Laves phase strengthening on the mechanical properties of high Cr ferritic steels for solid oxide fuel cell interconnect application. *Mater. Sci. Eng. A* **528,** 5888–5899 (2011).

60. Yamamoto, Y. *et al.* Alumina-forming austenitic stainless steels strengthened by laves phase and MC carbide precipitates. *Metall. Mater. Trans. A Phys. Metall. Mater. Sci.* **38 A,** 2737–2746 (2007).

61. Zeumert, B. & Sauthoff, G. Intermetallic NiAl□Ta alloys with strengthening Laves phase for high-temperature applications. I. Basic properties. *Intermetallics* **5,** 563–577 (1997).

62. Schirra, J. J., Caless, R. H. & Hatala, R. W. The effect of laves phases on the mechanical properties of wrought and cast+HIP Inconel 718. *Superalloys 718, 625 and Various Derivatives.* (ed. Loria, E. A.) 375–388 (The Minerals, Metals and Materials Society, 1991)

63. Ruiz-Yi, B. *et al.* The Different Roles of Entropy and Solubility in High Entropy Alloy Stability. *ACS Comb. Sci.* **18,** 596–603 (2016).
